# Biased agonism in psychopharmacology: an opportunity to improve efficacy and safety of treatments

**DOI:** 10.1017/S109285292510045X

**Published:** 2025-08-12

**Authors:** Gia Han Le, Sabrina Wong, Stavroula Bargiota, Swainson Jennifer, Heidi K.Y. Lo, Diana Orsini, Kayla Teopiz, Hernan F. Guillen-Burgos, Poh Khuen Lim, Roger S. McIntyre

**Affiliations:** 1 Poul Hansen Family Centre for Depression, University Health Network, Toronto, ON, Canada; 2 Brain and Cognition Discovery Foundation, Toronto, ON, Canada; 3 Institute of Medical Science, Faculty of Medicine, University of Toronto, Toronto, ON, Canada; 4 Department of Pharmacology & Toxicology, University of Toronto, Toronto, ON, Canada; 5 Department of Psychiatry, Aristotle University of Thessaloniki, Thessaloniki, Greece; 6 Department of Psychiatry, Faculty of Medicine, University of Alberta, Edmonton, AB, Canada; 7 Neuroscience and Mental Health Institute, University of Alberta, Edmonton, AB, Canada; 8 Department of Psychiatry, School of Clinical Medicine, LKS Faculty of Medicine, The University of Hong Kong, Hong Kong; 9 Pontificia Universidad Javeriana, Department of Psychiatry and Mental Health, Hospital Universitario San Ignacio, Bogota DC, Colombia; 10 Faculty of Medicine, Center for Clinical and Translational Research, Universidad El Bosque, Bogota DC, Colombia; 11 Universidad Simon Bolivar, Center for Clinical and Translational Research, Barranquilla, Colombia; 12 Department of Psychiatry, University of Toronto, Toronto, ON, Canada

**Keywords:** biased agonism, agonism, antagonism, psychopharmacology, efficacy, safety, GPCR, G-protein coupled receptors, GLP-1, psychedelics

## Abstract

G protein-coupled receptors (GPCRs) are involved in many physiological and pathophysiological processes. Conventional pharmacological models categorize the typology of pharmacologic ligands as agonists or antagonists. Biased agonism is a relatively newer pharmacodynamic characteristic that has potential to optimize therapeutic efficacy while minimizing adverse effects in psychiatric and neurological treatments. We conducted a narrative literature review of articles obtained from PubMed, Embase, and MEDLINE from inception to April 2025, focusing on pharmacologic antagonism (i.e., competitive, noncompetitive, uncompetitive) and agonism (i.e., full, partial, inverse, superagonism, biased). Primary and secondary articles defining these concepts were included, provided they addressed pharmacologic (rather than chemical) antagonism and agonism. Distinct mechanisms of antagonism and agonism were identified, each contributing nuanced receptor modulation beyond the conventional models. Notably, biased agonism facilitates targeted intracellular signaling (e.g., G protein- versus β-arrestin–mediated). Use cases demonstrate relatively greater efficacy (e.g., incretin receptor agonist, tirzepatide) and improved safety (e.g., serotonergic psychedelics, opioids). Biased agonism provides a potential avenue for future drug development, with emerging preclinical evidence suggesting potential to differentially activate intracellular pathways and thereby improve efficacy and safety profiles of psychopharmacologic agents—pending clinical validation. Future research vistas should aim to rigorously assess the long-term outcomes of biased agonism, explicitly addressing individual variability in receptor signaling and therapeutic response.

## Introduction

G protein-coupled receptors (GPCRs) are large transmembrane proteins composed of seven transmembrane domains.^1^ GPCRs share common yet diverse signal transduction mechanisms which are implicated in physiology, pathophysiology, and pharmacology.^1^^,^^2^ The heterogeneity in signal transduction effects related to GPCRs introduces complexity as well as opportunity for pharmacological discovery and development.^1^^,^^3^ It is currently estimated that over 35% of Food and Drug Administration (FDA)-approved pharmacologic agents target GPCRs, with over 150 GPCRs currently identified as being druggable.^2^^,^^4^

Pharmacological ligands (i.e., endogenous or synthetic ligands) bind to the GPCR extracellular domains, consequently triggering intracellular cascades which have implications for the treatment of disease processes.^1^^,^^5^^,^^6^ Notwithstanding the complexity of GPCR signaling, conventional pharmacological models have typically reduced the typology of pharmacologic ligands as either an agonist (i.e., a ligand that bind and activates a receptor to promote a conformational change that increases receptor-mediated signaling) or antagonists (i.e., a ligand that binds to without activating the receptor thus blocking/diminishing the effect of an agonist).^3^^,^^6^^–^^8^ While the agonist–antagonist paradigm is well characterized, other activities at GPCRs and subsequent effects on GPCR signaling cannot be parsimoniously reduced to agonism/antagonism.^6^

Emerging preclinical and/or preliminary evidence suggests that certain pharmacologic ligands, conventionally classified as agonists, can selectively (i.e., in a biased manner) activate a specific GPCR signaling pathway as opposed to contemporaneously activating multiple signaling cascades.^7^^,^^9^^–^^12^ Notwithstanding, clinical validation of these findings remains necessary. Consequently, pharmacologic ligands exerting biased agonism have the potential to enhance beneficial therapeutic outcomes (e.g., greater weight loss with incretin receptor agonists and neuroplastic changes) while avoiding the activation of pathways that mediate adverse drug reactions (e.g., psychedelic experiences, nausea).^1^^,^^6^^,^^12^

The overarching aim herein is to not only provide a critical evaluation but also a rationale for exploring whether biased agonism could serve as a theoretical framework to guide future pharmacological discoveries and developments, with potentially improved efficacy and/or safety, for the treatment of psychiatric conditions. It is of note that while compelling preclinical data exists, caution is warranted in extrapolating these findings to clinical populations until robust human evidence becomes available.

## Methods

We conducted a narrative review of articles published from inception to April, 2025. A search was conducted on literature databases including PubMed, Embase, and MEDLINE databases. The following search string was utilized for the search of relevant articles in the foregoing databases: (“agonist” OR “agonism” OR “full agonism” OR “partial agonism” OR “inverse partial agonist” OR “superagonism” OR “biased agonism” OR “antagonism” OR “antagonism” OR “competitive antagonism” OR “noncompetitive antagonism” OR “uncompetitive antagonism” OR “partial antagonism” OR “functional antagonism”). Furthermore, efficacy and safety examples of biased agonists and their effect on the discovery and development of pharmacologic agents were also searched.

The following eligibility criteria were employed during the screening process conducted by two independent reviewers (G.H.L. and S.W.). Primary research articles including human, animal, and in vitro studies were included. Secondary articles including, but not limited to, systematic reviews and meta-analyses were only included to define and characterize the different types of agonism and antagonism. Conclusions and findings from the foregoing secondary articles were not included when outlining preliminary evidence in support of a pharmacological agents’ efficacy or tolerability. Only articles referring to nonphysical/chemical antagonists were included. Furthermore, articles focused solely on chemical structure, unrelated to intracellular signaling or therapeutic implications, were excluded.

A purposive selection strategy was used to highlight representative agents, for each pharmacological ligand typology, that highlight mechanistic diversity and translational relevance in psychopharmacology. Article selection was informed by citation frequency, mechanistic clarity and clinical relevance. Due to the inherent flexibility of a narrative approach, no formal quality assessment or data synthesis was undertaken. Notwithstanding, to maintain rigor, we included all evidence when discussing efficacy, safety and tolerability of the representative agents included.

## Pharmacologic antagonism and agonism: definitions

### Antagonism

Pharmacologic antagonism is defined as a process wherein a pharmacologic agent binds to and inhibits the actions of a native agonist via interactions at a common receptor.^3^^,^^13^^,^^14^ There are three main principle mechanisms wherein pharmacologic agents antagonize native ligand activity at the receptor: competitive, noncompetitive, and uncompetitive antagonism.

#### Competitive antagonism

Competitive antagonists are pharmacologic agents that compete with a native ligand without activation of the receptor. Consequently, subsequent GPCR-mediated activation of signal transduction cascades is reduced and/or abrogated.^15^^,^^16^ Competitive antagonists are categorized as either competitive reversible or competitive irreversible antagonists.

Competitive reversible antagonists compete with a native ligand to bind to its canonical receptor. The occupancy of the receptor is a function of both the pharmacologic agent’s concentration and its affinity to the receptor.^17^^–^^19^ There are a finite number of receptor sites, which implies that as the concentration of the pharmacologic antagonist increases, there is a greater formation of antagonist–receptor complexes and a reduction in agonist–receptor complexes.^14^^,^^18^^,^^19^ In addition, a higher number of antagonist–receptor complexes would be expected as a function of higher receptor affinity by the pharmacologic antagonist. However, if the agonist concentration is sufficiently increased, the agonist can outcompete the antagonist for receptor binding to elicit maximal response (E_max_).^14^

For example, olanzapine is an atypical antipsychotic that is a competitive, reversible antagonist at various receptor sites including, but not limited to, dopamine (D_2_) and serotonin 5-HT_2A_ receptors.^20^ By binding noncovalently to the foregoing receptors, olanzapine prevents endogenous ligands (e.g., dopamine and serotonin) from binding, which reduces excessive dopaminergic and serotonergic signaling, contributing to its therapeutic effects in schizophrenia and bipolar depression.^21^

Competitive irreversible antagonism refers to a scenario wherein the pharmacologic antagonist competes with the native agonist for the target receptor; however, the robustness of the intermolecular interaction effectively confers antagonism insofar as the covalent bonds formed between the agent and the ligand are irreversible.^14^^,^^22^ Consequently, the effects of competitive irreversible antagonists will remain constant irrespective of endogenous agonist levels.^14^

#### Noncompetitive antagonism

Noncompetitive antagonism refers to the process wherein the pharmacologic antagonist does not directly compete with the native agonist for the identical binding site; however, it will impair the ability of an agonist to bind or to activate the receptor through steric and/or allosteric mechanisms.^14^^,^^23^ Steric (orthosteric) noncompetitive antagonism typically involves the antagonist binding to the same site as the agonist, consequently blocking activation via an irreversible or covalent interaction.^14^ Specifically, the antagonist remains bound to the receptor thus removing the receptor from the pool of receptors available for activation by an agonist. Allosteric noncompetitive antagonism occurs when the antagonist binds to a different (allosteric) site on the receptor, from the orthosteric site, consequently changing the receptor’s conformation to prevent activation by the agonist.^23^^,^^24^

Ketamine is an allosteric noncompetitive N-methyl-d-aspartate receptor (NMDAR) antagonist.^25^^,^^26^ Blockade of NMDARs on γ-aminobutyric acid (GABA)-ergic inhibitory interneurons by ketamine leads to disinhibition of pyramidal cells, resulting in a glutamate surge.^25^^,^^27^ Although ketamine does not occupy the glutamate-binding site and therefore does not prevent glutamate from binding to the orthosteric site on NMDARs, it binds within the receptor’s ion channel pore. This interaction prevents ion flow and impedes the activation of GABAergic interneurons.^14^^,^^25^^,^^28^ Glutamate binds to and activates postsynaptic α-amino-3-hydroxy-5-methyl-4-isoxazolepropionic acid receptors (AMPARs), which is believed to play a key role in ketamine’s antidepressant effects.^25^^,^^29^^,^^30^

#### Uncompetitive antagonism

Similar to noncompetitive antagonism, uncompetitive antagonism also involves an agonist that binds to an allosteric site on the receptor, separate from an agonist’s binding site; however, antagonist–receptor binding only occurs postreceptor activation, during which the receptor pore is open.^31^^,^^32^

Memantine, FDA-approved in the treatment of moderate-to-severe Alzheimer’s disease, is an uncompetitive antagonist of NMDARs.^33^^,^^34^ Rather than binding at the orthosteric site, memantine preferentially binds within the open, activated ion channel and blocks excessive calcium influx.^34^^,^^35^ Memantine’s blockade of calcium influx reduces excitotoxicity in Alzheimer’s disease while still allowing for physiological neurotransmission to occur.^36^^,^^37^ Separately, dextromethorphan is also an uncompetitive antagonist and modulates glutamate signaling of NMDARs.^38^ The binding of dextromethorphan to activated NMDARs results in the inhibition of excessive excitatory neurotransmission, and ultimately reduced excitotoxicity and disrupted synaptic plasticity, which may contribute to depressive symptoms in depressive disorders.^38^

### Agonism

In select disease states, a physiological system may be insufficiently active, providing the basis for pharmacological agents to increase activity of the system.^3^ The foregoing pharmacological agents exhibit receptor agonism wherein binding to the receptor results in receptor activation.^39^ There are different types of receptor agonism, including full, partial, inverse, and superagonism. Another type of receptor agonism is biased agonism, which will be discussed in a separate section.

#### Full agonism

Full agonism refers to a substance or agent that mimics the effects of an endogenous ligand.^7^ In this case, the agent binds to the orthosteric binding site and activates the physiological system to the same degree as the endogenous ligand, a maximal response.^40^ Consequently, pharmacological agents that bind to a receptor and activate it to produce a biological response, mimicking the maximal response induced by an endogenous ligand (e.g., neurotransmitter), are widely used in pain management and have high addiction potential.^41^

Morphine is an example of a full agonist which binds to and activates μ-opioid receptors (MORs) to induce profound analgesia—a property that, while therapeutically valuable, also underlies its significant side-effect profile.^42^^–^^44^ Separately, methadone (MTD) is also a full MOR agonist. Racemic methadone ((R,S)-MTD) consists of two enantiomers, (R)-MTD and (S)-MTD, wherein both exhibit full MOR agonism to produce analgesia; however, they differ in their abuse potential.^45^ Recent evidence indicates that compared to (R)-MTD, (S)-MTD does not robustly stimulate the dopaminergic reward pathway in the ventral tegmental area (VTA); therefore, exhibiting lower reinforcing efficacy in rats.^45^ In contrast, (R)-MTD exhibits greater efficacy on dopaminergic signaling activation and was associated with reliable self-administration in rats.^45^ The findings indicate that the abuse liability of (R,S)-MTD is mediated by (R)-MTD instead of (S)-MTD.^45^ The foregoing phenomenon highlights that while full MOR agonism is often associated with elevated abuse liability, differences in agonist–receptor interactions at specific brain regions may modulate the risk profile of different full agonists.

#### Partial agonism

Similar to full agonism, partial agonism also refers to a pharmacological agent that binds to the orthosteric site on the receptor. Partial agonists activate the receptor to increase the activity of the system, but only with partial efficacy compared to a full agonist or the endogenous ligand that elicits a maximal response.^46^ This approach can be advantageous when a specific physiological outcome needs to be controlled, as seen with certain antipsychotics (e.g., aripiprazole, brexpiprazole, cariprazine) or pain medication (e.g., buprenorphine).^47^^–^^51^

For example, the partial agonism of aripiprazole at dopamine and serotonin receptors allows for the balancing of neurotransmitter activity in both hyper and hypodopaminergic states.^52^^–^^54^ The dual action of aripiprazole addresses both positive and negative symptoms in schizophrenia as well as both depressive and manic poles of bipolar disorder and may lead to fewer side effects than are common with traditional antipsychotics.^53^^,^^55^^,^^56^

#### Inverse agonism

The observation that receptors may be activated in the absence of a native ligand led to the discovery of pharmacologic agents that can reduce constitutive receptor activity. Costa and Herz (1989) conducted a study of wild type, endogenously expressed delta opioid receptors in NG108-15 neuroblastoma cell membranes, and found that several ligands, previously thought to be antagonists, decreased GTPase activity stimulated by these receptors.^57^ Since their effects opposed those of agonists, these ligands were classified as inverse agonists.

While agonists are characterized by intrinsic efficacy, or the ability to enhance receptor activity, inverse agonists show negative intrinsic activity. Similar to how the intrinsic efficacy of agonists varies depending on their structure, leading to distinctions between strong and weaker (partial) agonists, the negative intrinsic efficacy of inverse agonists can also be characterized as strong or weak (partial) inverse agonists.^7^

A range of antipsychotic medications exert their therapeutic effects through antagonism at dopamine D2 and serotonin 5-HT2A receptors. The role of the 5-HT2A receptor in the pathophysiology of psychosis has been underscored by the psychotomimetic effects of serotonergic hallucinogens such as LSD or psylocybin, that act as agonists at the 5-HT2A receptor.^58^^,^^59^ This observation provided the basis for the hypothesis that 5-HT2A antagonism could be a viable target for antipsychotic development.^60^ However, clinical trials involving selective 5-HT2A antagonists—notably volinanserin—failed to demonstrate sufficient efficacy in schizophrenia populations, leading to the discontinuation of such compounds in late-stage development.^61^

More recently, the 5-HT_2A_ inverse agonist pimavanserin (Nuplazid) was FDA approved in the treatment of Parkinson’s disease (PD) psychosis.^62^ Pimavanserin acts as an inverse agonist and antagonist at serotonin 5-HT_2A_ receptors (Ki 0.087 nM) and 5-HT_2C_ receptors (Ki 0.44 nM). Primavanserin exhibits low binding to sigma 1 receptors (Ki 120 nM) and negligible affinity (Ki > 300 nM) for 5-HT_2B_, dopaminergic (i.e., D2), muscarinic, histaminergic, adrenergic receptors, and calcium channels.^63^ Unlike typical antipsychotics, pimavanserin does not interfere with dopaminergic signaling pathways, which poses an advantage for individuals vulnerable to motor side effects.^63^^,^^64^ Notwithstanding, by acting as an inverse agonist at 5-HT_2A_Rs, primavanserin reduces phosphoinositide signaling thus downregulating 5-HT_2A_-driven excitatory signaling to dampen psychotic symptoms (e.g., hallucinations and delusions) associated with PD psychosis.^65^

#### Superagonism

Superagonism refers to the phenomenon wherein a ligand not only activates a receptor but can also induce a greater maximal effect than endogenous ligands or full agonists.^66^^,^^67^ This occurs when the ligand’s intrinsic efficacy is greater than that of endogenous neurotransmitters or hormones; therefore, superagonists are able to activate the receptor to a functional level that surpasses what occurs normally under physiological conditions.^66^^,^^68^

Notwithstanding the efficacy of superagonists, it is also associated with greater risk of side effects or potential receptor desensitization as a result of overstimulation of targeted pathways.^69^^,^^70^ For example, isotonitazene is a synthetic opioid that is a superagonist of μ-opioid receptors (MORs). Compared to other opioids such as morphine, hydromorphone and fentanyl, isotonitazene demonstrates greater MOR signaling efficacy; therefore, it exhibits greater potency and overall efficacy for reducing pain (isotonitazene > fentanyl; *F*(1,26) = 8.25, p = 0.008).^71^^,^^72^ It is of note that preclinical findings from extant literature also indicate that isotonitazene’s superagonism is associated with greater and prolonged respiratory depression than fentanyl.^71^ The foregoing example underscores the importance of balancing therapeutic potency and safety considerations, which highlights a broader principle relevant to biased agonism wherein targeted efficacy must be carefully weighed against potential adverse outcomes and their severities.

### Biased agonism

**Figure 1.**
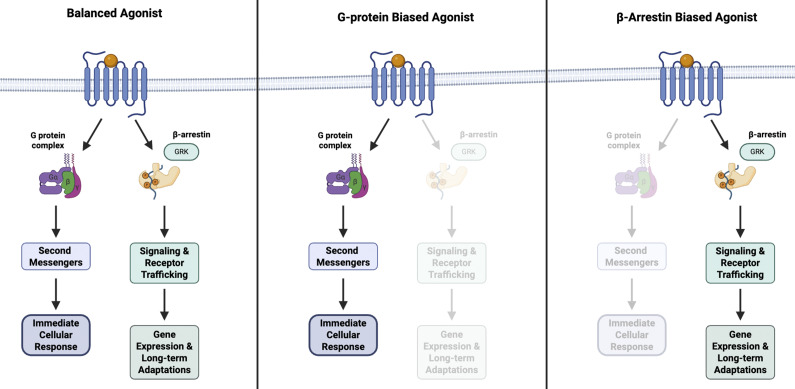
G-protein-coupled receptor-biased agonism. Left: Balanced agonist that activates both the G-protein- and β-arrestin-mediated pathways. Middle: G-protein-biased agonist that selectively activates the G-protein intracellular pathway. Right: βarrestin-biased agonist that selectively activates the β-arrestin intracellular pathway. Created in BioRender. Le, G. (2025). https://BioRender.com/3tmy5ld.

Contemporary mathematical and pharmacological studies indicate that GPCRs are highly dynamic with the ability of adopting multiple structural conformations and signaling states.^73^^,^^74^ Depending on the ligand that binds to the GPCR, both G protein-mediated and β-arrestin pathways may be activated or one pathway may be preferentially stimulated over the other (Figure 1).^75^^,^^76^ Preferential activation of a particular pathway is known as biased agonism, also referred to as functional selectivity.^9^ Biased and selective agonism are distinct, wherein the latter refers to receptor selectivity rather than preferential pathway activation after a ligand binds.^8^ This phenomenon allows for a more targeted modulation of distinct intracellular responses and treatment of clinical symptoms.^9^^,^^77^^–^^79^ Biased agonism is a well-established principle in GPCR research and various GPCR families have been studied including, serotonergic, opioid, adrenergic, cannabinoid, muscarinic, and metabotropic glutamate receptors.^12^^,^^80^^–^^82^

Extant literature indicates that ligands exhibiting biased agonism may be a novel avenue to achieve targeted symptom relief while minimizing additional side effects. For example, current data indicate that functionally selective ligands at opioid receptors are able to achieve pain relief without the normally associated abuse liability, dysphorigenic properties, or psychomimetic components of ligands that traditionally bind at these sites.^83^^,^^84^ In addition, recent pharmacological advances indicate that engaging D_2_R arrestin-biased signaling via GSK3β or 5HT_2A_ arrestin-biased Src/Akt signaling may enable more targeted treatments for schizophrenia, psychosis, and other mood disorders.^85^^–^^87^ Preclinical evidence suggests that biased agonism may present a novel strategy for potentially enhancing both therapeutic efficacy and safety to achieve better patient tolerability. However, robust clinical studies are essential to confirm these hypothesized advantages in patient populations.

## Opportunity for enhancing efficacy with biased agonism

Preclinical studies suggest that regulating downstream effectors such as GSK3β may contribute to achieving antipsychotic effects without impairing motor function.^85^^–^^87^ In psychiatry, biased ligands targeting dopamine D2 receptors—selectively activating arrestin-dependent pathways—have been explored to decrease extrapyramidal symptoms primarily induced by conventional antipsychotics. While promising in preclinical settings, rigorous clinical validation remains to be necessary to determine real-world applicability.

Biased agonism has also shown potential in metabolic and neuroendocrine contexts. Glucagon-like peptide-1 receptor agonists (GLP-1 RAs) are widely prescribed for treating type 2 diabetes mellitus (T2DM) and obesity.^88^ Preliminary evidence from secondary analyses and observational studies suggests that GLP-1 RAs may exert indirect benefits for mood, cognitive, and substance-related clinical outcomes, largely through improvements in metabolic parameters (e.g., weight management and glycemic modulation).^89^^–^^93^ However, clinical trials in psychiatric populations directly measuring the foregoing clinical symptom outcomes at endpoints independent of metabolic changes are necessary to confirm these potential effects. Notwithstanding, there remain concerns regarding suicidality among persons treated with GLP-1 RAs; however, evidence of causality has not been established.^94^ Evidence from extant literature indicates that metabolic disturbances, including obesity and untreated/poorly maintained diabetes, may predispose individuals to and exacerbate psychiatric symptoms (e.g., depression, anxiety), which emphasizes the importance of careful monitoring.^95^^,^^96^ Recent evidence in older adults and animal models indicates that GLP-1 RAs exert antidepressant effects independent of glycemic control.^97^^,^^98^ Supporting the foregoing findings, Gunturu et al. (2024) similarly highlighted the promise of GLP-1 RAs for psychiatric treatment, specifically in improving mood and cognitive functioning.^99^ It is hypothesized that the therapeutic effects that GLP-1s may exert across dimensions of psychopathology are a consequence of their ability to target neurobiological systems relevant to neuroplasticity and neuroprotection.^100^ Notwithstanding the preliminary findings in support of GLP-1 receptor agonists’ potential in psychiatric contexts, evidence of antidepressant efficacy and precognitive effects independent of metabolic improvements is currently limited, highlighting the need for focused clinical studies.

**Figure 2.**
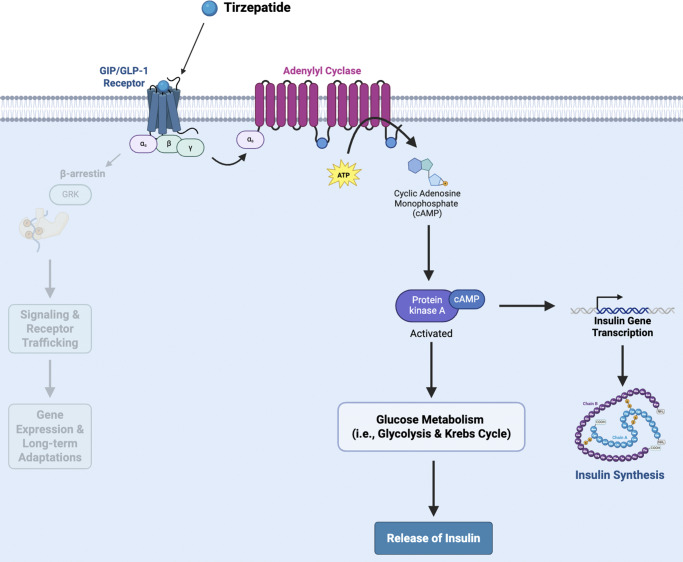
Tirzepatide’s hypothesized mechanism of action. Once bound to the GIP/GLP-1 receptor, tirzepatide selectively activates the G-protein intracellular pathway, specifically the Gɑs-protein-mediated signaling pathway. This ligand–receptor interaction results in receptor conformational change and activation. Subsequently, the activated receptor exchanges the guanosine diphosphate (GDP) on the ɑs-subunit to guanosine triphosphate (GTP), which activates the G-protein, leading to the dissociation of the ɑ-subunit. The activated ɑs-subunit binds to and stimulates the activation of its effector protein adenylyl cyclase (AC). Activated AC catalyzes the conversion of adenosine triphosphate (ATP) to cyclic adenosine monophosphate (cAMP). cAMP will then bind to and activate protein kinase A (PKA), which (1) activates glucose metabolism to increase the release of insulin and (2) initiates insulin gene transcription for insulin synthesis.^136^ Created in BioRender. Le, G. (2025). https://BioRender.com/ce23rn8.

Building upon this therapeutic foundation, tirzepatide, a dual glucose-dependent insulinotropic polypeptide (GIP) and GLP-1 receptor agonist exhibiting biased signaling, has demonstrated superior efficacy in weight reduction and glycemic control compared to standard GLP-1 agonists.^101^^–^^103^ Extant literature reports that while tirzepatide’s primary indication is for the treatment of T2DM, its biased activation of distinct intracellular pathways may also benefit psychiatric populations with metabolic comorbidities.^98^^,^^104^^,^^105^ The precise mechanisms underlying tirzepatide’s potential effects on mood and cognitive functioning remain currently unclear and require focused clinical investigation. Notwithstanding, current hypotheses propose that biased agonism at the GLP-1Rs, favoring cyclic adenosine monophosphate (cAMP) over β-arrestin signaling, may be a key mechanism underlying its superior efficacy in metabolic improvements (Figure 2).^106^^–^^108^ By improving metabolic health, which has been reported to be associated with mood and cognitive disturbances in extant literature, tirzepatide’s biased agonism may represent a promising approach, with potentially reduced risk of adverse events (e.g., nausea and vomiting), for individuals with metabolic disorders.^105^^,^^109^ However, any clinical psychiatric benefits (e.g., improvements in mood or cognitive functioning) have yet to be explicitly demonstrated to be independent of metabolic changes. Therefore, further research is required to elucidate tirzepatide’s mood and cognitive effects in persons with comorbid T2DM or obesity and psychiatric disorder. While evidence for tirzepatide’s full impact on the foregoing parameters are still emerging, it is posited that tirzepatide’s biased agonism may underscore its superior efficacy in improving therapeutic indices across metabolic and psychiatric conditions compared to other GLP-1 RAs.

## Opportunity for enhancing safety with biased agonism

By selectively activating intracellular pathways, biased agonism can potentially minimize off-target effects associated with adverse outcomes while enhancing therapeutic benefits.^9^^,^^110^^,^^111^ Oliceridine (TRV130), is a G protein-biased mu-opioid receptor agonist able to produce analgesic efficacy and at the same time reduce adverse effects associated with beta-arrestin engagement.^112^ Initial clinical trials suggest that oliceridine may maintain analgesic efficacy with reduced hypoventilation compared to morphine; however, its long-term safety and efficacy as well as in comparison with traditional opioids remain to be conclusively demonstrated in larger-scale and long-term clinical trials.^113^ In addition, it has also been demonstrated in rodent models that cannabinoid CB_1_ receptor-biased agonists are able to reduce sedation and psychomotor impairment and still maintain therapeutic efficacy.^114^

Separately, there is uncertainty about whether hallucinogenic experiences mediate the antidepressant effects of serotonergic psychedelics (e.g., psilocybin).^12^^,^^115^^,^^116^ Preliminary evidence suggests that coadministration of 5-HT_2A_ antagonist not only prevents psychedelic experiences with psilocybin but does not appear to interfere with antidepressant efficacy.^117^ Research efforts are attempting to determine whether fully antagonizing 5-HT_2A_ activity or biased agonism of the 5-HT_2A_ which aims to block the hallucinogenic effects associated with the activation of G_qɑ_ protein signaling pathway is capable of antidepressant effects in the absence of a psychedelic experience.^12^^,^^118^^,^^119^

Biased agonism endeavors for psilocybin could similarly target 5-HT_2A_ pathways to minimize the profound hallucinogenic “trip.” By selectively activating downstream intracellular pathways hypothesized to underlie its therapeutic benefits and minimizing the activation of signalling pathways associated with perceptual distortions, drug tolerability would improve.^12^^,^^58^ Notwithstanding, the foregoing potential mechanisms are currently theoretical as the ability to fully separate therapeutic from hallucinogenic effects, via biased signaling, remains to be demonstrated in clinical contexts.

**Figure 3.**
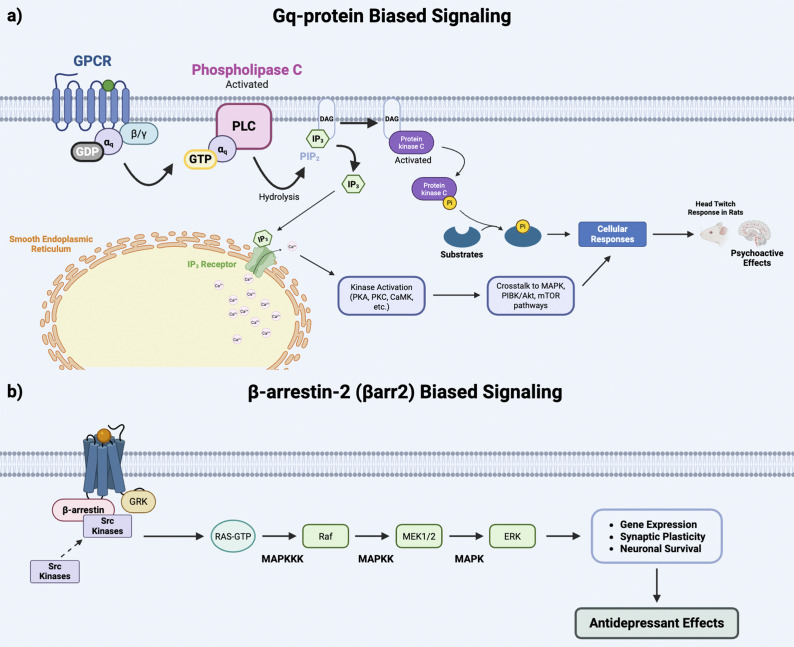
G-protein- versus β-arrestin-biased signaling. (a) Gq-protein-biased signaling. (b) β-arrestin-2 (βarr2)-biased signaling. Extant literature indicates βarr2-biased signaling produces antidepressant effects without psychoactive effects (ie, head twitch response in rats).^12^^,^^58^^,^^85^^,^^120^^,^^137^ Furthermore, it is observed that ERK and MAPK signaling are downregulated in individuals with depression, which suggests they may play a role in inducing antidepressant effects observed with psychedelics.^12^^,^^138^ Created in BioRender. Le, G. (2025). https://BioRender.com/fvcgyje.

Preclinical evidence from recent animal studies indicates that when only the β-arrestin-2 signaling pathway was engaged, no hallucinogenic effects were observed (i.e., head twitch response).^120^^,^^121^ In addition, 5-HT_2A_R biased agonism of the β-arrestin-2 signaling pathway was also associated with antidepressant effects in mice without producing psychoactive effects (Figure 3).^12^^,^^122^ The foregoing findings in animal models suggest 5-HT_2A_R biased agonism for the β-arrestin-2 pathway may mitigate the psychoactive effects, and ultimately reduce the toxicity associated with the “trip” as well as improve drug tolerability and safety while still providing beneficial therapeutic effects (Figure 3) (e.g., antidepressant, procognitive, etc.).

While biased agonism has demonstrated notable efficacy and safety benefits in preclinical models, translating these findings from in vitro and animal models to clinical populations remains uncertain and may present challenges due to fundamental differences in the complexity of human psychedelic experiences.^123^ The rodent head-twitch response only provides a behavioral model/strategy for inferring human hallucinogenic experiences; therefore, it may not capture some but not fully reflect the complex cognitive and affective components underlying the phenomenology of the human psychedelic experience.

In clinical research contexts, several ongoing clinical trials investigating psilocybin for treatment-resistant depression have employed risperidone—a 5-HT_2A_ antagonist—to block the hallucinogenic effects that result from activation of the G_qɑ_ protein signaling pathway, a strategy informed by well-established evidence from drug–drug interaction studies involving classical psychedelics.^12^^,^^118^^,^^119^ In this way, it can be determined whether hallucinogenic experiences are necessary for the facilitation of antidepressant effects. More investigations in human studies are underway. To conclude, psychedelic analogues are being created by scientists with the ability to maintain their therapeutic potential for mood disorders and decrease psychedelic effects by controlling biased signaling pathways. The contemporary research aims to develop a new generation of serotonergic drugs which could deliver fast and enduring antidepressant treatment without harming cognitive abilities or causing psychotic-like adverse reactions.

## Discussion

Herein, this narrative review synthesizes existing evidence, highlighting the potential evolution from conventional agonist–antagonist paradigms (i.e., “all-or-none” modulators) towards more nuanced pharmacological frameworks, notably biased agonism. The discovery of these ligand–receptor concepts have important implications for the development of psychiatric and neurological pharmacotherapeutics considering the degree and direction of receptor activation can be adjusted to maximize therapeutic benefit while minimizing adverse side effects (e.g., hallucinogenic effects, respiratory depression, etc.).^6^^,^^9^^,^^124^ Notwithstanding, it remains crucial to recognize the preliminary nature of the current body of evidence with regard to clinical translation.

Biased agonism (functional selectivity) has emerged as a promising approach to induce pathway-specific receptor signaling, thereby improving the therapeutic index—a ratio that compares the blood concentration at which a drug causes a therapeutic effect to the amount that causes death (in animal studies) or toxicity (in human studies).^125^ As aforementioned, the development of oliceridine (TRV130) shows how biased G protein-mediated signaling at the mu-opioid receptor is able to maintain analgesia while reducing respiratory depression associated with activating the β-arrestin pathway.^112^^,^^113^ In addition, CB_1_-receptor-biased agonism has also been demonstrated to preserve pain relief and anti-inflammatory effects in preclinical settings without the marked sedation or psychomotor impairment.^114^

### Clinical implications of biased agonism in psychiatry

Biased agonism represents a potential innovative strategy to transform psychiatric drug development, allowing selective modulation of intracellular signaling to engage the most beneficial intracellular downstream signaling cascades, thereby increasing therapeutic effects, while avoiding pathways associated with adverse effects.^125^ In psychiatry, this paradigm is especially relevant for disorders where existing treatments may produce metabolic disruption, unwanted sedation, and/or extrapyramidal symptoms (e.g., impaired motor control).^126^

Notwithstanding, it is important to note that several examples of biased agonism discussed herein are currently supported predominantly by preclinical, indirect, and/or metabolic-linked outcomes rather than direct psychiatric endpoint evidence. While the current body of literature consists of compelling preclinical rationales and limited clinical evidence, inferences of direct psychiatric benefits remain speculative and hypothesis-generating, requiring rigorous clinical confirmation.

Psychedelic compounds, such as psilocybin, have generated significant interest in the field of psychiatry due to preliminary evidence suggesting rapid antidepressant effects in persons with treatment-resistant depressive disorders; however, uncertainty remains regarding whether the therapeutic benefits can be decoupled from hallucinogenic effects via biased signaling mechanisms.^12^^,^^115^ As discussed, preclinical findings indicate that selectively activating the 5-HT_2A_R-linked β-arrestin-2 pathway may not only preserve the antidepressant effects but also reduce psychoactive effects in rodent depression-like models.^120^^–^^122^ Furthermore, preclinical studies are evaluating other biased agonists, specifically of 5-HT_1A_ receptors (e.g., NLX-101 and NLX-204), that display rapid-acting antidepressant properties and cognitive functioning benefits like ketamine without the dissociative side effects.^127^ Further research is necessary to determine whether the foregoing findings observed in animal models are maintained in human studies in terms of efficacy and safety. Furthermore, findings from recent research efforts with GLP-1 and the dual GIP/GLP-1 receptor agonists (i.e., tirzepatide) indicate potential metabolic benefits; however, indirect implications for psychiatric outcomes remain speculative wherein mood and cognitive benefits remain heterogeneous.^101^^,^^128^

Notably, biased agonists may exert different effects in discrete brain regions as a result of region-specific variations in signaling protein expression. For example, recent evidence indicates that a β-arrestin-2-biased D_2_R ligand may elicit opposing antagonistic and agonistic effects in the striatum and cortex, respectively.^123^ This phenomenon has also been observed with 5-HT_1A_R-biased agonists. For example, although not U.S. FDA approved yet, NLX-101 and NLX-112 both exhibit biased agonism at 5-HT_1A_Rs; however, they each exhibit differential properties and target distinct brain regions.^129^ Specifically, NLX-101 preferential activates cortical and brain stem 5-HT_1A_Rs and has been observed to be potently active in rodent models of depression and respiratory control.^129^ In contrast, NLX-112 exhibits prominent activation of 5-HT_1A_ autoreceptors in Raphe nuclei and motor-relevant pathways, and has shown promising activity in animal models of PD.^129^ The foregoing examples highlight region-specific nuances of biased agonism, which could be particularly valuable for different conditions (e.g., schizophrenia) wherein optimal treatment requires opposing or differential effects in different brain regions.

In psychiatry, several pressing unmet clinical needs remain unaddressed by existing pharmacotherapies such as treatment-resistant depression (TRD), cognitive dysfunction across affective and psychotic disorders, affective instability, medication-induced adverse events, as well as effectively and simultaneously addressing various clinical symptoms in highly comorbid disorders (e.g., depression and obesity). For example, as aforementioned, while interest has sparked for psilocybin’s rapid antidepressant effects, there remains debate in regard to its psychedelic effects and widespread applicability.^12^^,^^58^^,^^115^^,^^116^ In line with this, preclinical evidence suggests that a β-arrestin-2-biased D_2_R ligand may not only maintain the rapid-antidepressant effects without the psychedelic “trip.”^123^ Separately, 5-HT_1A_R-biased agonists (e.g., NLX-204, NLX-101) also show promise in producing rapid antidepressant effects akin to ketamine without inducing dissociation.^129^ Notwithstanding the lack of clinical evidence on the foregoing agents, preliminary preclinical findings suggest that they may be uniquely positioned to meet the clinical demand for not only fast-acting, but also well-tolerated treatments in TRD. Such agents, exhibiting biased agonism, hold the potential to transform psychiatric pharmacotherapy towards enhancing mechanistic precision and patient-centered outcomes.

### Future directions: emerging tools and frameworks

While the central aim of this narrative review is to critically evaluate the clinical and translational relevance of biased agonism for the potential development and improvement of psychiatric pharmacotherapy, it is of note that recent advances in ligand discovery have enabled the development of pathway-selective compounds. For example, tools such as structure–activity relationship (SAR) modeling have been applied to predict and optimize biased signaling profiles in the hopes of improving clinical symptom outcomes, especially for treatment-resistant mood disorders.^130^ Furthermore, quantitative models (e.g., ΔLog(Emax/EC50)) have also been proposed wherein drug discovery can apply the concept of biased ligand quantification and compare signaling bias across ligands in a large-scale, standardized manner.^131^ Although an in-depth review of these methodologies is beyond the scope of the present narrative, their continued development underscores the momentum of biased agonism as a translational development strategy.

### Limitations of translational evidence

Notwithstanding recent efforts, it is pertinent that robust future clinical trials specifically designed to investigate agents exhibiting biased agonism and how selectively engaging specific pathways, while inhibiting others downstream, translates to meaningful long-term improvements in clinical symptom outcomes.^123^^,^^132^^,^^133^ In addition, rigorous clinical trials are needed to evaluate the long-term safety profile of these agents.

Separately, given the novelty of biased agonism and complexity of GPCR signaling, the translation from preclinical to human clinical outcomes involves significant uncertainty. Heterogeneity across persons such as region-specific differences in receptor and effector molecule distributions, receptor dynamics, and genetic polymorphisms represent important translational barriers that must be systematically addressed in future research and initiatives. For example, individual genetic differences in receptor conformation or downstream effectors (e.g., G protein-coupled receptor kinases (GRK) polymorphism) may affect the degree to which a biased agonist reduces adverse effects or enhances therapeutic outcomes.^110^^,^^133^^–^^140^

## Conclusion

Biased agonism represents an innovative pharmacological concept with potential to improve safety profiles, and neurologic and psychiatric clinical outcomes by selectively modulating intracellular signaling pathways. Notwithstanding, substantial translational challenges remain which emphasizes the need for rigorous clinical validation and interdisciplinary collaboration—especially among pharmacologists, psychiatrists, and neuroscientists—to understand the complex relationships between the molecular signatures of GPCRs, and more importantly confirm the efficacy and safety in diverse patient populations. Understanding how different GPCR conformations turn cellular signaling into behavioral responses remains a key research focus in academia and industry. Discoveries from this field are expected to lead to the development of novel, safer, and more effective therapeutic strategies to improve clinical outcomes and quality of life in persons living with psychiatric disorders, especially in treatment-resistant populations.
